# Effects of Gut Microbiota on the Bioavailability of Bioactive Compounds from Ginkgo Leaf Extracts

**DOI:** 10.3390/metabo9070132

**Published:** 2019-07-05

**Authors:** Min Sun Choi, Jeon-Kyung Kim, Dong-Hyun Kim, Hye Hyun Yoo

**Affiliations:** 1 Institute of Pharmaceutical Science and Technology and College of Pharmacy, Hanyang University, Ansan, Gyeonggi-do 15588, Korea; 2 Neurobiota Research Center, Department of Life and Nanopharmaceutical Sciences and College of Pharmacy, Kyung Hee University, Seoul 02447, Koera

**Keywords:** gut microbiota, ginkgo leaf extracts, flavonoid, metabolism, pharmacokinetics

## Abstract

Ginkgo leaf extract (GLE) is a popular herbal medicine and dietary supplement for the treatment of various diseases, including cardiovascular disease. GLE contains a variety of secondary plant metabolites, such as flavonoids and terpenoids, as active components. Some of these phytochemicals have been known to be metabolized by gut microbial enzymes. The aim of this study was to investigate the effects of the gut microbiota on the pharmacokinetics of the main constituents of GLE using antibacterial-treated mice. The bilobalide, ginkgolide A, ginkgolide B, ginkgolide C, isorhamnetin, kaempferol, and quercetin pharmacokinetic profiles of orally administered GLE (600 mg/kg), with or without ciprofloxacin pretreatment (150 mg/kg/day for 3 days), were determined. In the antibacterial-treated mice, the maximum plasma concentration (C_max_) and area under the curve (AUC) of isorhamnetin were significantly (*p* < 0.05) increased when compared with the control group. The C_max_ and AUC of kaempferol and quercetin (other flavonol glycosides) were slightly higher than those of the control group, but the difference was not statistically significant, while both parameters for terpenoids of GLE showed no significant difference between the antibacterial-treated and control groups. These results showed that antibacterial consumption may increase the bioavailability of isorhamnetin by suppressing gut microbial metabolic activities.

## 1. Introduction

Herbal products contain a range of bioactive phytochemicals (e.g., polyphenols and terpenes) [1,2,3]. The use of herbal products as dietary supplements has increased dramatically in recent years, and most are taken daily for the prevention and treatment of lifestyle-related chronic diseases [4,5,6,7,8,9]. Orally administered phytochemicals may be biotransformed by the gut microbiota before absorption from the gastrointestinal tract [10,11]. Many research papers have demonstrated that the gut microbiota is involved in the biotransformation of natural active components such as flavonoids, saponins, phenylethanoid glycosides, and alkaloids, both in vitro and in vivo [12,13,14,15,16]. Such metabolic reactions by the gut microbiota may contribute to the activation or detoxification of phytochemicals. For example, orally administered Rb1 and baicalin are metabolized into ginsenoside Rd/compound K and baicalein, respectively, by gut microbiota [17,18,19,20].

Ginkgo leaf extract (GLE) is made from the leaves of the ginkgo biloba tree. GLE is one of the most popular herbal products worldwide. It is taken daily over a long period, either as a herbal medicine or as a dietary supplement, mostly for the prevention and treatment of cardiovascular disease [21,22]. It is also taken for the treatment of asthma and bronchitis [23,24]. The major bioactive components of GLE are flavonoids and terpene lactones [25,26,27,28]. The flavonoids in GLE are mainly present in a glycoside form, including kaempferol, quercetin, and isorhamnetin, linked to monoglycosides, diglycosides, or triglycosides by a β-glycosidic bond. The major terpene lactones include diterpenes ginkgolides A, B, and C and sesquiterpene bilobalide. After oral administration of GLE, terpene lactones are absorbed in the gastrointestinal tract in the parent form, but such absorption may be poor for unchanged flavonol glycosides [25,29,30]. The glycosides are broken down into their absorbable aglycones by glycosidase from the gut microbiota, which are then absorbed and further conjugated to glucuronides or sulfates by host phase II enzymes [10,13,14].

However, despite the potential effects of the gut microbial metabolism on the pharmacokinetics and biological actions of ginkgo flavonols, few studies are available that focus on these effects for GLE, and the effects of gut microbial metabolism on ginkgo terpene lactones have not yet been fully characterized. There is substantial recent evidence that the composition diversity and enzyme activity of the gut microbiota might fluctuate in response to external factors such as antibiotics [31,32,33,34,35,36]. For example, ciprofloxacin, which generally has little activity against standard cultivated anaerobes, affects the gut microbiota composition [32,34,35]. Dethlefsen et al. discovered that five days of antibacterial ciprofloxacin administration impacted the levels of about 33% of the bacterial taxa in the gut and diminished the taxonomic richness within days of initial exposure [37]. Thus, if active components (terpene lactones and flavonols) in GLE endure biotransformation by gut microbiota, their absorption within the digestive tract may well be altered by antibacterial treatment, which could lead to changes in their pharmacological potency and eventually result in unexpected side effects.

Many studies have examined the effects of gut microbiota composition modifiers such as antibiotics and probiotics on the pharmacokinetics of phytochemicals or drugs. For example, alterations in the metabolic activities of the gut microbiota by antibiotic treatment led to alteration of the pharmacokinetics of hesperidin in rats [33]. Treatment with ampicillin caused alteration of the gut microbiota composition to modulate the pharmacokinetics of aspirin in rats [38]. In addition, a recent study demonstrated that the intake of probiotics may influence the absorption of orally administered drugs through the disturbance of gut-microbiota-mediated drug metabolism [39].

**Figure 1:**
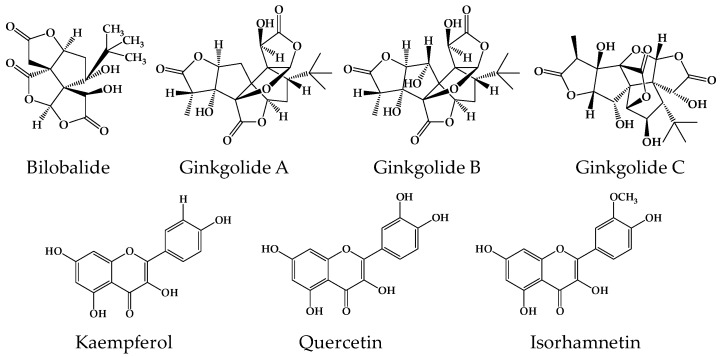
The chemical structures of the ginkgo terpene lactones and flavonols investigated in this study.

Therefore, in the present study, changes in the pharmacokinetic pattern of ginkgo terpene lactones and flavonols (Figure 1) following antibacterial treatment were investigated to understand the role of gut bacterial metabolism in the bioavailability of GLE components using antibacterial-exposed animals. Ciprofloxacin was selected as a model antibacterial agent since it is an orally administered, broad-spectrum, and frequently prescribed antibacterial agent.

## 2. Results

### 2.1. Analytical Methods for Gingko Terpene Lactones and Flavonols

**Figure 2:**
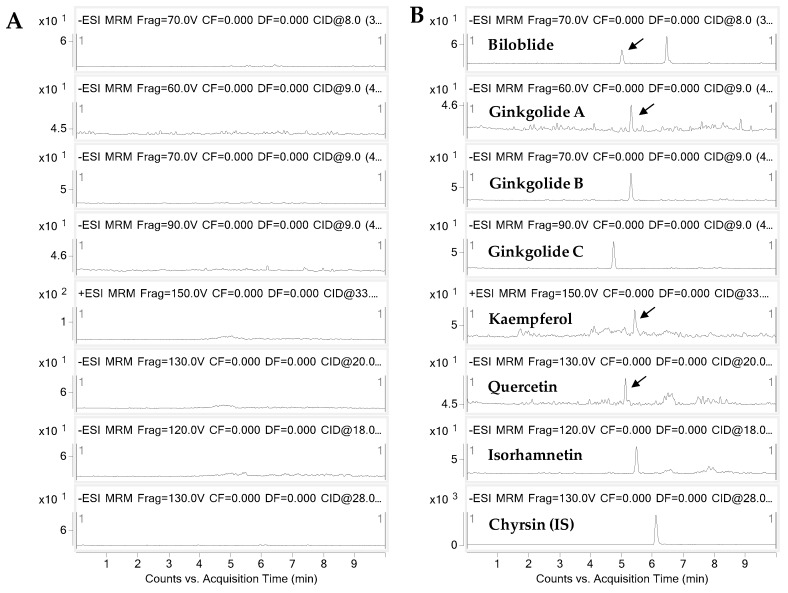
Representative extracted ion chromatograms of (**A**) blank and (**B**) spiked mouse plasma at 5 ng/mL.

The bioanalytical method for the determination of gingko terpene lactones and flavonols in mouse plasma was developed using liquid chromatography–tandem mass spectrometry (LC-MS/MS). The representative extracted ion chromatograms are shown in Figure 2. The plasma calibration curves were constructed using six calibration standards. The correlation coefficients of the calibration curves were greater than 0.99, and the accuracies and precisions at each concentration were all satisfactory (<15%, data not shown). The calibration equations, linear ranges, limits of detection (LODs), and limits of quantification (LOQs) for the seven components are summarized in Table 1.

### 2.2. Pharmacokinetic Assessment of Terpene Lactones in Control and Antibacterial-Treated Mice

**Figure 3:**
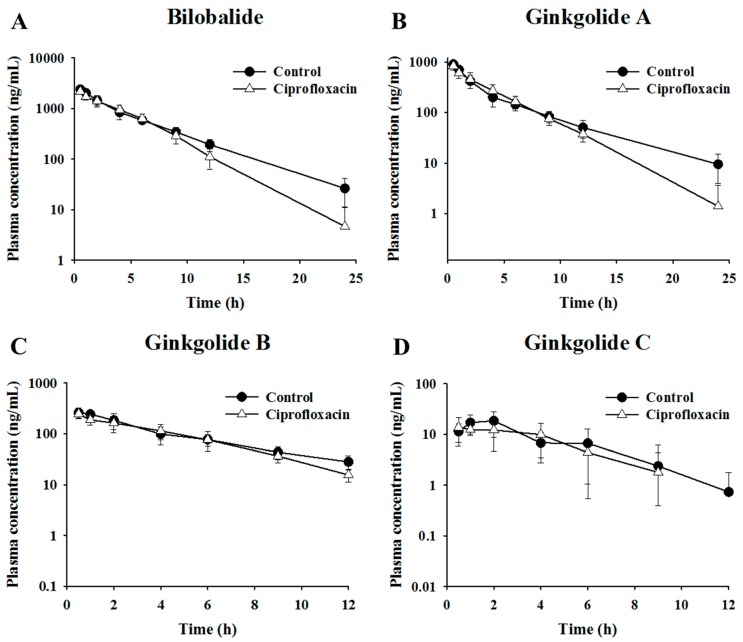
The profiles of the mean plasma concentration of terpene lactones over time after administration of 600 mg/kg ginkgo leaf extract in control and ciprofloxacin-treated mice. Mean plasma concentration–time profiles of (**A**) bilobalide (**B**) ginkgolide A, (**C**) ginkgolide B, and (**D**) ginkgolide C.

The present study investigated the possibility of herb–drug interactions between terpene lactones (bilobalide, ginkgolide A, ginkgolide B, and ginkgolide C) and antibacterials (ciprofloxacin) associated with altered metabolic activities of the gut microbiota. The mean plasma concentration–time profiles of bilobalide, ginkgolide A, ginkgolide B, and ginkgolide C after oral administration of GLE in the control and antibacterial-treated mice are shown in Figure 3. The pharmacokinetic parameters are summarized in Table 2. The differences in the maximum plasma concentration (C_max_) and area under the curve (AUC) between the control and antibacterial-treated groups were not statistically significant. These results suggest that the gut microbiota may not influence the intestinal metabolism and absorption of major terpene lactones in GLE.

### 2.3. Pharmacokinetic Assessment of Flavonols in Control and Antibacterial-Treated Mice

**Figure 4:**
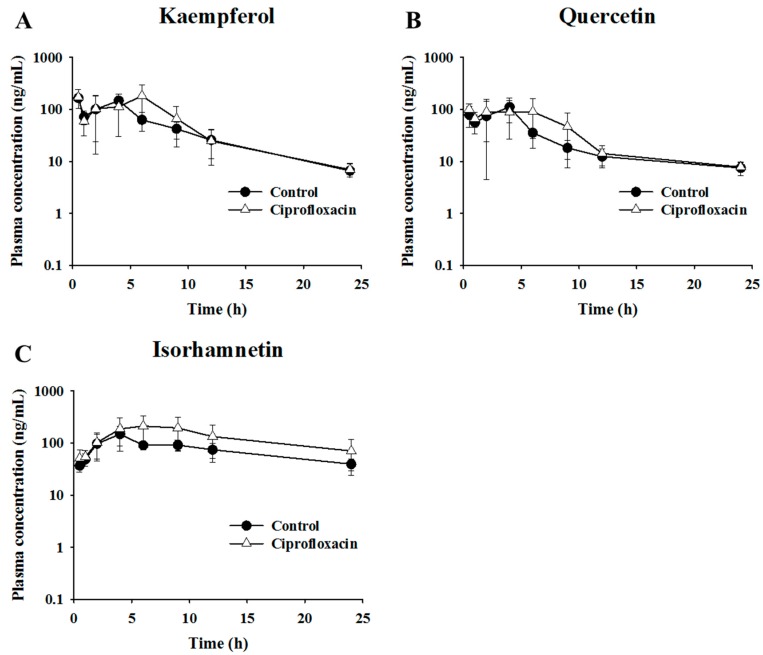
The profiles of mean plasma concentration of flavonols over time after administration of 600 mg/kg ginkgo leaf extract in control and ciprofloxacin-treated mice. Mean plasma concentration–time profiles of (**A**) kaempferol (**B**) quercetin, and (**C**) isorhamnetin.

The effects of intestinal microflora were examined to verify the pharmacokinetic behaviors of flavonol glycoside after oral administration of GLE in ciprofloxacin-treated mice. As the ginkgo flavonols should circulate in various forms, such as glucuronides, sulfates, glycosides, or aglycones, the major ginkgo flavonols—kaempferol, quercetin, and isorhamnetin—were measured as aglycones after acid hydrolysis. The mean plasma concentration–time profiles of isorhamnetin, kaempferol, and quercetin after oral administration of GLE in the control and ciprofloxacin-treated mice are shown in Figure 4. The pharmacokinetic parameters are summarized in Table 3. The AUC values for kaempferol in the control and ciprofloxacin-treated mice were 1103.3 ± 341.1 ng·h/mL and 1397.0 ± 509.0 ng·h/mL, respectively. The AUC values for quercetin in the control and ciprofloxacin-treated mice were 773.1 ± 210.3 ng·h/mL and 934.0 ± 275.1 ng·h/mL, respectively. For kaempferol and quercetin, the C_max_ and AUC of the ciprofloxacin-treated group were slightly higher than for the control group, but the difference was not statistically significant. For isorhamnetin, the C_max_ values in the control and ciprofloxacin-treated mice were 156.0 ± 56.4 ng/mL and 268.1 ± 102.8 ng/mL, respectively, and the AUC values were 1798.4 ± 497.5 ng·hr/mL and 3143.7 ± 1613.5 ng·hr/mL, respectively. The C_max_ and AUC of the ciprofloxacin-treated group were significantly higher than those of the control group (*p* < 0.05). The mean AUC of isorhamnetin was increased by 1.7 times in ciprofloxacin-treated mice. These data therefore indicate that the uptake of isorhamnetin was increased by antibacterial treatment.

### 2.4. Effects of Ciprofloxacin on the Composition of Gut Microbiota

**Figure 5:**
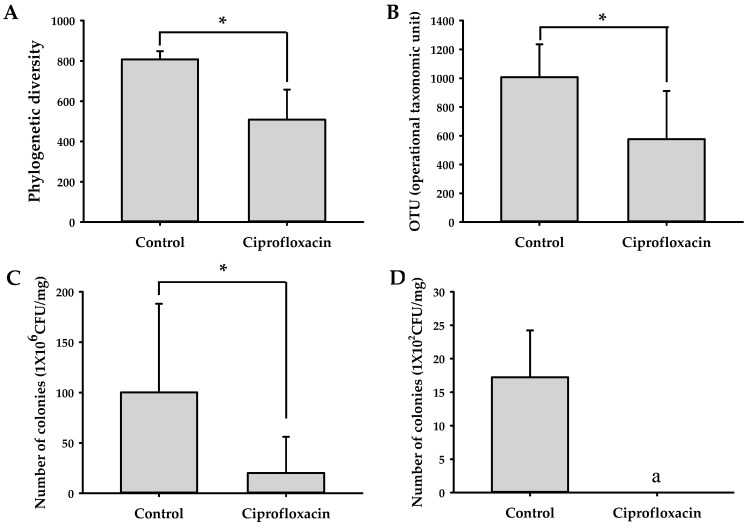
Effect of ciprofloxacin on the growth of the gut microbiota: (**A**) OTUs and (**B**) phylogenic diversity of the fecal microbiome from control and ciprofloxacin-treated mice (*n* = 3); the number of colonies grown in (**C**) blood liver (BL) and (**D**) deoxycholate hydrogen sulfide lactose (DHL) agar plates. * *p* < 0.05 vs. control. ^a^ Colony was not observed.

The profile of the gut microbiota in control and ciprofloxacin-treated mice was analyzed by pyrosequencing of the bacterial 16S rRNA fragments with fecal samples. The numbers of operational taxonomic units (OTUs) in each group are shown in Figure 5. The OTU value was lower in ciprofloxacin-treated mice than in control mice. The phylogenetic diversity was also reduced in ciprofloxacin-treated mice. Furthermore, when mouse stools were cultured in blood liver (BL) and deoxycholate hydrogen sulfide lactose (DHL) agar plates, the number of total cultured colonies was suppressed by ciprofloxacin treatment. These results indicate that ciprofloxacin administration decreased the number and diversity of the gut microbiota. 

## 3. Discussion

The present study investigated the influence of the gut microbiota on the pharmacokinetics of terpene lactones and flavonols in GLE. For this, an antibacterial-treated mice model was used to cause fluctuation in the gut microbiota composition and profile. The data suggested that the pharmacokinetics of ginkgo flavonols may be affected by alteration of the gut microbiota caused by orally administered ciprofloxacin. Isorhamnetin, in particular, showed a significant increase in systemic exposure following antibacterial treatment.

A previous study reported that the in vitro biotransformation rates and residence times of the 14 bioactive components of GLE clearly differed due to intestinal bacteria between normal, diabetic, and diabetic nephropathy rats [16]. Tang et al. found that the metabolic activities of intestinal bacteria in diabetic nephropathy rats differed markedly from those in normal rats, which consequently influenced the biotransformation of GLE [16]. Generally, the half-life of terpene lactones was longer in the intestinal bacteria matrix of diabetic rats than in normal rats. However, in the present study, we could not observe any significant differences in the pharmacokinetics of terpene lactones. Interestingly, in the case of flavonoids, the metabolism of some components was suppressed, but that of others was enhanced by the intestinal bacteria of the disease state. These findings suggest that gut microbial enzymes may be engaged in the metabolic reactions of flavonoids in different ways, depending on the substance.

It is known that only a small portion of the flavonoid glycosides consumed (<10%) is absorbed in the gastrointestinal tract [40]. This reveals the considerable role of the gut microbiota in the biotransformation of flavonoids [10]. According to the literature, gut microbial enzymes may eliminate glycosides, glucuronides, and sulfates of flavonoids to produce flavonoid aglycones, which are further biotransformed by gut microbial enzymes to produce a variety of ring fission products [10]. For example, quercetin glycosides may be metabolized by gut microbial glucosidases to yield quercetin, from which gut microbiota may generate ring fission products such as 3,4-dihydroxyphenylacetic acid, 3-hydroxyphenylacetic acid, protocatechuic acid, and hippuric acid [10,14].

Lin et al. researched the effect of the gut microbiota on the biotransformation of quercetin, kaempferol, naringenin, apigenin, and luteolin in mice [14]. They affirmed the metabolic ability of intestinal microbiota using an in vitro fermentation model with fecal suspension, and they assessed the pharmacokinetics of these flavonoids in control and antibiotic-treated mice after flavonoid consumption. The serum flavonoid levels were comparable in both groups, but the phenolic metabolite concentrations were lower in the antibiotic-treated mice than in the control mice. They concluded that the intestinal microbiota is required for the biotransformation of flavonoids but may not influence the bioavailability of flavonoids. These results were partly consistent with our results, based on the data for kaempferol and quercetin. However, our data suggest that the bioavailability of some flavonoids (e.g., isorhamnetin) may be affected by the gut microbiota.

This would be different to how the gut microbiota influences the pharmacokinetics of flavonoids, depending on the metabolic reactions predominantly mediated by gut microbial enzymes. In the case of isorhamnetin, the ring cleavage reactions might have been primarily suppressed by antibacterial treatment, and, consequently, the absorption of isorhamnetin may have been increased. Another possible pathway is *O*-demethylation of the methoxy group. This can be supported by the fact that only isorhamnetin has the methoxy group among the three flavonoids investigated. Meanwhile, our previous study demonstrated that the absorption of hesperidin—a citrus flavanone glycoside—was decreased by antibiotic treatment [33]. This was explained by a decrease in gut microbial glucosidase metabolic activities. Such conflicting results with flavonoids could be understood in the same context as the in vitro biotransformation results for GLE components reported by Tang et al. (described above) [16]. Therefore, more in-depth study of the biotransformation of flavonoids by the gut microbiota should be undertaken to better understand its role in the bioavailability of flavonoids.

The limitation of this study is that the details of mechanisms involved in the alteration of the pharmacokinetics of isorhamnetin following antibacterial treatment were not elucidated. Ciprofloxacin may affect the physiological conditions of the gastrointestinal tract, such as tight junctions or transports, to cause the alteration of isorhamnetin pharmacokinetics. According to the paper by Duan et al., the transport of isorhamnetin is a complicated process involving passive diffusion, paracellular and transcellular pathways, and polarized transport mechanisms mediated by influx and efflux transporters [41]. In addition, only isorhamnetin showed a significant change in AUC among the three flavonols. Therefore, we concluded that the alteration of gut microbial metabolic activities by ciprofloxacin might be a more influential factor than the changes to the physiological conditions of the gastrointestinal tract.

## 4. Materials and Methods 

### 4.1. Chemicals

Ciprofloxacin, bilobalide, ginkgolide A, ginkgolide B, ginkgolide C, isorhamnetin, kaempferol, quercetin, chrysin, ascorbic acid, and hydrogen chloride were purchased from Sigma Chemicals (St. Louis, MO, USA). Formic acid was purchased from Merck KGaA (Germany). HPLC-grade acetonitrile and ethyl acetate were purchased from J.T. Baker (Phillipsburg, NJ, USA).

### 4.2. Preparation of Ginkgo Leaf Extract

GLE was prepared according the method of Schwabe [42]. Briefly, dry ginkgo biloba leaves (100 g), which were purchased from Gyeongdong herb market (Seoul, Korea), were pulverized, extracted with 750 mL of 60% acetone with agitation for 30 min (57–59 °C), and filtrated. The residual fraction was re-extracted under the same conditions. The combined extracts were concentrated under reduced pressure to a solid content of 30% to 40%; water was added; and the extract was left to cool (12 °C) with stirring before finally being centrifuged. Ammonium sulfate (30%) was added to the resulting supernatant, and liquid–liquid extraction was performed twice using a methylethylketone and acetone mixture (6:4 to 1:1 ratio). The methylethylketone/acetone fraction was concentrated under reduced pressure to a solid content of 50%; water was added to a solid content of 10%; and the fraction was stirred three times with water-saturated *n*-butanol. The combined butanol fraction was concentrated under reduced pressure to a solid content of 50%, diluted with water and ethanol, and stirred with *n*-heptane three times. The water fraction was concentrated to a solid content of 50% and dried at 80 °C. The dried powder was used as GLE. The contents of bilobalide, ginkgolide A, ginkgolide B, ginkgolide C, isorhamnetin, kaempferol, and quercetin in the ginkgo leaf extract were 0.23, 0.12, 0.12, 0.05, 18.8, 32.6, and 31.9 mg/g, respectively. The total flavonol glycoside concentration was determined as follows: C_total flavonoids_ = 2.504C_quercetin_ + 2.588C_kaempferol_ + 2.437C_isorhamnetin_, where Cχ stands for the concentration of χ. The total flavonol glycoside content was 209 mg/g [25,43,44].

### 4.3. Animal Experiments

This animal experimental protocol was approved by the Committee for the Care and Use of Laboratory Animals in the College of Pharmacy, Hanyang University, and performed in accordance with the National Institutes of Health and Hanyang University Guides for Laboratory Animal Care and Use (HY-2018-011A).

Male C57BL/6 mice weighing 22–25 g were supplied by Orient Bio. (Songnam, Korea). The mice were maintained in air-conditioned animal quarters at 23 ± 2 °C and 55% ± 10% relative humidity with a controlled 12 h light/dark cycle and free access to food and water. They were randomly divided into two groups as control and antibacterial-treated mice. For preparation of pseudo-germ-free animals, mice were orally administered ciprofloxacin (150 mg/kg) once per day for 3 days. The pharmacokinetic study was carried out 3 days after the final administration. GLE (600 mg/kg) was administered orally for both the control and pseudo-germ-free groups. Whole blood samples (40 μL) were collected serially from the orbital vein at 0.5, 1, 2, 4, 6, 9, 12, and 24 h after drug administration. The blood samples were centrifuged at 13,200 rpm for 5 min and plasma was collected.

### 4.4. Blood Sample Preparation

To 20 μL plasma samples, 10 μL 10% ascorbic acid and 4 M hydrogen chloride 20 μL were added. The samples were incubated for 1 h at 90 °C. After incubation, 600 μL ethyl acetate containing 1000 ng/mL chrysin (internal standard; IS) was added and vortexed. Then, the samples were centrifuged at 13,200 rpm for 5 min, and the supernatants were evaporated to dryness under an N_2_ stream at 50 °C. The residue was dissolved in 100 μL acetonitrile, and a 5 μL aliquot was injected into the LC-MS/MS system for analysis.

### 4.5. LC-MS/MS Conditions

The LC-MS/MS system consisted of an Agilent 1260 Infinity HPLC system with an Agilent 6460 triple-quadrupole mass spectrometer equipped with an electrospray ionization source (Agilent Technologies, Palo Alto, CA, USA). The column used for the separation was a Poroshell120 EC-C8 (3.0 × 100 mm, 2.7 μm; Agilent Technologies) and was maintained at 40 °C. The mobile phase was composed of 0.1% formic acid in distilled water (A) and 0.1% formic acid in 90% acetonitrile (B). The elution program was set as 10%–90% B at 0–4 min, maintained for 2 min, and followed by 4 min re-equilibration to the initial condition over 0.1 min. 

The mass spectrometer was operated in positive ion electrospray mode. Quantification was obtained using the multiple reaction monitoring (MRM) acquisition mode by monitoring the precursor ion to product ion transitions of *m*/*z* 325 > 163 for bilobalide, *m*/*z* 453 > 351 for ginkgolide A, *m*/*z* 423 > 367 for ginkgolide B, *m*/*z* 439 > 383 for ginkgolide C, *m*/*z* 315 > 300 for isorhamnetin, *m*/*z* 287 > 153 for kaempferol, and *m*/*z* 301 > 151 for quercetin and for chrysin. The capillary voltage was set at 3500 kV and the source temperature was set to 350 °C. Nitrogen was used as a drying gas at 300 °C and 10 L/min.

### 4.6. Preparation of Stock Solutions and Calibration Standards

Stock solutions of bilobalide, ginkgolides A, ginkgolides B, ginkgolides C, isorhamnetin, kaempferol, quercetin, and chrysin were dissolved with methanol to 1 mg/mL. Working standard solutions were prepared by diluting the stock solution with methanol to final concentrations ranging from 0.05 μg/mL to 3 μg/mL for bilobalide, isorhamnetin, kaempferol, and quercetin and from 0.05 μg/mL to 1 μg/mL for ginkgolide A, ginkgolide B, and ginkgolide C. The working solution of IS was diluted with ethyl acetate to a final concentration of 1000 ng/mL. These working standard solutions (100 μL) were added to blank mouse plasma to yield calibration standards of 5–3000 ng/mL for bilobalide, isorhamnetin, kaempferol, and quercetin and of 5–1000 ng/mL for ginkgolide A, ginkgolide B, and ginkgolide C. A weighted linear regression model was used to construct the calibration curves. The weighting factors used were 1/*x* for bilobalide, ginkgolide B, ginkgolide C, kaempferol, and isorhamnetin and 1/*x*^2^ for ginkgolide A and quercetin.

### 4.7. Culture and Pyrosequencing of Gut Microbiota

Mouse fecal samples were collected 72 h after the final treatment with ciprofloxacin from control and antibacterial-treated mice. The fecal samples were diluted in general anaerobic medium broth (Nissui Pharm Inc., Tokyo, Japan), inoculated in BL and DHL agar plates, and incubated anaerobically (for 2 days) or aerobically (for 1 day) at 37 °C. In addition, the fecal samples were subjected to pyrosequencing on a 454 GS FLX Titanium Sequencing System (Roche, Branford, CT, USA). Sequence reads were identified using the EzTaxon-e database (http://eztaxon-e.ezbiocloud.net/) based on the 16S rRNA sequence data. 

### 4.8. Data Analysis

The pharmacokinetic behaviors of seven components of GLE were investigated using WinNonlin 5.3 (Pharsight, St. Lousi, MO, USA). The total area under the plasma concentration–time curve to the end time (AUC), the maximum plasma concentration (C_max_), and the time to reach C_max_ (T_max_) were estimated by non-compartmental analysis of the plasma concentration versus time. Statistical analysis among the control and the co-administered antibiotics groups were carried out via Student’s *t*-test, with a *p* value less than 0.05 attributed to statistical significance.

## Figures and Tables

**Table 1:** Calibration equations, linear ranges, LODs, and LOQs for the components in ginkgo leaf extracts.

Components	Linear Range (ng/mL)	Slope	Intercept	R	LOD ^a^ (ng/mL)	LOQ ^b^ (ng/mL)
Bilobalide	5–3000	0.5043	0.0020	0.9952	2	5
Ginkgolide A	5–1000	0.0866	0.0006	0.9937	2	5
Ginkgolide B	5–1000	0.3158	0.0036	0.9990	2	5
Ginkgolide C	5–1000	0.2241	0.0032	0.9982	2	5
Kaempferol	5–3000	0.0070	−0.0009	0.9963	3	5
Quercetin	5–3000	0.1918	−0.0012	0.9928	2	5
Isorhamnetin	5–3000	0.6044	−0.0010	0.9977	2	5

^a^ LOD, the limit of detection, was determined based on S/N = 3. ^b^ LOQ, the limit of quantitation, was determined based on S/N = 10 and accuracy and precision <20%.

**Table 2:** Pharmacokinetic parameters of terpene lactones after oral administration of ginkgo leaf extract in control and ciprofloxacin-treated mice.

Components	Control Group (*n* = 7)			Antibacterial-Treated Group (*n* = 7)		
	T_max_ (h)	C_max_ (ng/mL)	AUC (ng·h/mL)	T_max_ (h)	C_max_ (ng/mL)	AUC (ng·h/mL)
Bilobalide	0.5 ± 0.0	2388.6 ± 249.0	10692.7 ± 708.3	0.5 ± 0.0	2170.7 ± 251.2	9639.5 ± 945.2
Ginkgolide A	0.5 ± 0.0	902.6 ± 101.5	3047.8 ± 204.2	0.5 ± 0.0	831.7 ± 134.8	2950.3 ± 462.8
Ginkgolide B	0.7 ± 0.3	271.8 ± 52.8	1314.6 ± 321.8	0.5 ± 0.0	247.9 ± 43.8	1063.2 ± 213.5
Ginkgolide C	1.6 ± 0.6	20.4 ± 8.2	82.4 ± 40.2	1.6 ± 1.2	17.4 ± 6.9	63.7 ± 33.0

**Table 3:** Pharmacokinetic parameters of flavonols after oral administration of ginkgo leaf extract in control and ciprofloxacin-treated mice.

Components	Control Group (*n* = 7)			Antibacterial-Treated Group (*n* = 7)		
	T_max_ (h)	C_max_ (ng/mL)	AUC (ng h/mL)	T_max_ (h)	C_max_ (ng/mL)	AUC (ng·h/mL)
Kaempferol	1.9 ± 1.6	191.6 ± 42.1	1103.3 ± 341.1	2.8 ± 2.3	235.3 ± 71.7	1397.0 ± 509.0
Quercetin	2.4 ± 1.6	140.9 ± 48.4	773.1 ± 210.3	3.8 ± 1.9	165.1 ± 40.9	934.0 ± 275.1
Isorhamnetin	3.7 ± 0.8	156.0 ± 56.4	1798.4 ± 497.5	5.3 ± 2.1	268.1 ± 102.8	3143.7 ± 1613.5

## References

[1] Crozier A., Jaganath I.B., Clifford M.N. (2009). Dietary phenolics: Chemistry, bioavailability and effects on health. Nat. Prod. Rep..

[2] Formica J.V., Regelson W. (1995). Review of the biology of Quercetin and related bioflavonoids. Food Chem. Toxicol..

[3] Manach C., Scalbert A., Morand C., Remesy C., Jimenez L. (2004). Polyphenols: Food sources and bioavailability. Am. J. Clin. Nutr..

[4] Amiot M.J., Riva C., Vinet A. (2016). Effects of dietary polyphenols on metabolic syndrome features in humans: A systematic review. Obes. Rev..

[5] Bischoff S.C. (2008). Quercetin: Potentials in the prevention and therapy of disease. Curr. Opin. Clin. Nutr. Metab. Care..

[6] Bjorklund G., Chirumbolo S. (2017). Role of oxidative stress and antioxidants in daily nutrition and human health. Nutrition.

[7] Hun Lee J., Shu L., Fuentes F., Su Z.Y., Tony Kong A.N. (2013). Cancer chemoprevention by traditional chinese herbal medicine and dietary phytochemicals: Targeting nrf2-mediated oxidative stress/anti-inflammatory responses, epigenetics, and cancer stem cells. J. Tradit. Complement. Med..

[8] Rathee P., Chaudhary H., Rathee S., Rathee D., Kumar V., Kohli K. (2009). Mechanism of action of flavonoids as anti-inflammatory agents: A review. Inflamm. Allergy Drug Targets.

[9] Siasos G., Tousoulis D., Tsigkou V., Kokkou E., Oikonomou E., Vavuranakis M., Basdra E.K., Papavassiliou A.G., Stefanadis C. (2013). Flavonoids in atherosclerosis: An overview of their mechanisms of action. Curr. Med. Chem..

[10] Murota K., Nakamura Y., Uehara M. (2018). Flavonoid metabolism: The interaction of metabolites and gut microbiota. Biosci. Biotechnol. Biochem..

[11] Wang L., Ravichandran V., Yin Y., Yin J., Zhang Y. (2019). Natural Products from Mammalian Gut Microbiota. Trends Biotechnol..

[12] Fan L., Zhao X., Tong Q., Zhou X., Chen J., Xiong W., Fang J., Wang W., Shi C. (2018). Interactions of Dihydromyricetin, a Flavonoid from Vine Tea (Ampelopsis grossedentata) with Gut Microbiota. J. Food Sci..

[13] Kawabata K., Yoshioka Y., Terao J. (2019). Role of Intestinal Microbiota in the Bioavailability and Physiological Functions of Dietary Polyphenols. Molecules.

[14] Lin W., Wang W., Yang H., Wang D., Ling W. (2016). Influence of Intestinal Microbiota on the Catabolism of Flavonoids in Mice. J. Food Sci..

[15] Liu Z., Bruins M.E., Ni L., Vincken J.P. (2018). Green and Black Tea Phenolics: Bioavailability, Transformation by Colonic Microbiota, and Modulation of Colonic Microbiota. J. Agric. Food Chem..

[16] Tang D., Yu Y., Zheng X., Wu J., Li Y., Wu X., Du Q., Yin X. (2014). Comparative investigation of in vitro biotransformation of 14 components in Ginkgo biloba extract in normal, diabetes and diabetic nephropathy rat intestinal bacteria matrix. J. Pharm. Biomed. Anal..

[17] Chen H., Li Z., Li Y.J., Wu X.W., Wang S.R., Chen K., Zheng X.X., Du Q., Tang D.Q. (2015). Simultaneous determination of baicalin, oroxylin A-7-O-glucuronide and wogonoside in rat plasma by UPLC-DAD and its application in pharmacokinetics of pure baicalin, Radix Scutellariae and Yinhuang granule. Biomed. Chromatogr..

[18] Kang M.J., Ko G.S., Oh D.G., Kim J.S., Noh K., Kang W., Yoon W.K., Kim H.C., Jeong H.G., Jeong T.C. (2014). Role of metabolism by intestinal microbiota in pharmacokinetics of oral baicalin. Arch. Pharm. Res..

[19] Kim K.A., Yoo H.H., Gu W., Yu D.H., Jin M.J., Choi H.L., Yuan K., Guerin-Deremaux L., Kim D.H. (2014). Effect of a soluble prebiotic fiber, NUTRIOSE, on the absorption of ginsenoside Rd in rats orally administered ginseng. J. Ginseng Res..

[20] Kim K.A., Yoo H.H., Gu W., Yu D.H., Jin M.J., Choi H.L., Yuan K., Guerin-Deremaux L., Kim D.H. (2015). A prebiotic fiber increases the formation and subsequent absorption of compound K following oral administration of ginseng in rats. J. Ginseng Res..

[21] Haines D.D., Bak I., Ferdinandy P., Mahmoud F.F., Al-Harbi S.A., Blasig I.E., Tosaki A. (2000). Cardioprotective effects of the calcineurin inhibitor FK506 and the PAF receptor antagonist and free radical scavenger, EGb 761, in isolated ischemic/reperfused rat hearts. J. Cardiovasc. Pharmacol..

[22] Kim M.S., Bang J.H., Lee J., Han J.S., Baik T.G., Jeon W.K. (2016). Ginkgo biloba L. extract protects against chronic cerebral hypoperfusion by modulating neuroinflammation and the cholinergic system. Phytomedicine.

[23] Jeong H.S., Kim K.H., Lee I.S., Park J.Y., Kim Y., Kim K.S., Jang H.J. (2017). Ginkgolide A ameliorates non-alcoholic fatty liver diseases on high fat diet mice. Biomed. Pharmacother..

[24] Tao Z., Jin W., Ao M., Zhai S., Xu H., Yu L. (2019). Evaluation of the anti-inflammatory properties of the active constituents in Ginkgo biloba for the treatment of pulmonary diseases. Food Funct..

[25] Chen F., Li L., Xu F., Sun Y., Du F., Ma X., Zhong C., Li X., Wang F., Zhang N. (2013). Systemic and cerebral exposure to and pharmacokinetics of flavonols and terpene lactones after dosing standardized Ginkgo biloba leaf extracts to rats via different routes of administration. Br. J. Pharmacol..

[26] Ding S., Dudley E., Plummer S., Tang J., Newton R.P., Brenton A.G. (2006). Quantitative determination of major active components in Ginkgo biloba dietary supplements by liquid chromatography/mass spectrometry. Rapid Commun. Mass Spectrom..

[27] Stromgaard K., Nakanishi K. (2004). Chemistry and biology of terpene trilactones from Ginkgo biloba. Angew. Chem. Int. Ed. Engl..

[28] Van Beek T.A., Montoro P. (2009). Chemical analysis and quality control of Ginkgo biloba leaves, extracts, and phytopharmaceuticals. J. Chromatogr. A.

[29] Aa L., Fei F., Tan Z., Aa J., Wang G., Liu C. (2018). The pharmacokinetics study of ginkgolide A, B and the effect of food on bioavailability after oral administration of ginkgolide extracts in beagle dogs. Biomed. Chromatogr..

[30] Yan-Yan Z., Li-Li G., Guo-Ming S., Rong R., Jing-Zhen T. (2016). Determination of Ginkgolides A, B, C, J and Bilobalide in Plasma by LC-ESI (-)/MS/MS (QQQ) and its Application to the Pharmacokinetic Study of Ginkgo Biloba Extract in Rats. Drug Res. (Stuttg.).

[31] Becattini S., Taur Y., Pamer E.G. (2016). Antibiotic-Induced Changes in the Intestinal Microbiota and Disease. Trends Mol. Med..

[32] Dudek-Wicher R.K., Junka A., Bartoszewicz M. (2018). The influence of antibiotics and dietary components on gut microbiota. Prz. Gastroenterol..

[33] Jin M.J., Kim U., Kim I.S., Kim Y., Kim D.H., Han S.B., Kim D.H., Kwon O.S., Yoo H.H. (2010). Effects of gut microflora on pharmacokinetics of hesperidin: A study on non-antibiotic and pseudo-germ-free rats. J. Toxicol. Environ. Health A.

[34] Modi S.R., Collins J.J., Relman D.A. (2014). Antibiotics and the gut microbiota. J. Clin. Investig..

[35] Perez-Cobas A.E., Gosalbes M.J., Friedrichs A., Knecht H., Artacho A., Eismann K., Otto W., Rojo D., Bargiela R., Von Bergen M. (2013). Gut microbiota disturbance during antibiotic therapy: A multi-omic approach. Gut.

[36] Yoo H.H., Kim I.S., Yoo D.H., Kim D.H. (2016). Effects of orally administered antibiotics on the bioavailability of amlodipine: Gut microbiota-mediated drug interaction. J. Hypertens..

[37] Dethlefsen L., Huse S., Sogin M.L., Relman D.A. (2008). The pervasive effects of an antibiotic on the human gut microbiota, as revealed by deep 16S rRNA sequencing. PLoS Biol..

[38] Kim I.S., Yoo D.H., Jung I.H., Lim S., Jeong J.J., Kim K.A., Bae O.N., Yoo H.H., Kim D.H. (2016). Reduced metabolic activity of gut microbiota by antibiotics can potentiate the antithrombotic effect of aspirin. Biochem. Pharmacol..

[39] Kim J.K., Choi M.S., Jeong J.J., Lim S.M., Kim I.S., Yoo H.H., Kim D.H. (2018). Effect of Probiotics on Pharmacokinetics of Orally Administered Acetaminophen in Mice. Drug Metab. Dispos..

[40] Duda-Chodak A., Tarko T., Satora P., Sroka P. (2015). Interaction of dietary compounds, especially polyphenols, with the intestinal microbiota: A review. Eur. J. Nutr..

[41] Duan J., Xie Y., Luo H., Li G., Wu T., Zhang T. (2014). Transport characteristics of isorhamnetin across intestinal Caco-2 cell monolayers and the effects of transporters on it. Food Chem. Toxicol..

[42] Schwabe W. (1994). Method of Preparation of An Extract from Gingo Biloba Leaves and Pharmaceuticals Containing the Extract. U.S. Patent.

[43] Gray D., LeVanseler K., Pan M.D. (2005). Determination of flavonol aglycones in Ginkgo biloba dietary supplement crude materials and finished products by high-performance liquid chromatography: Single laboratory validation. J. AOAC. Int..

[44] Zun H., Yunan Z., Chao W., Weiyu W., Dongming X. (2010). Acid Hydrolytic Method for determination of ginkgo biloba total flavonoids in rat plasma by HPLC for pharmacokinetic studies. Tsinghua Sci. Technol..

